# A Panel of Genetic Polymorphism for the Prediction of Prognosis in Patients with Early Stage Non-Small Cell Lung Cancer after Surgical Resection

**DOI:** 10.1371/journal.pone.0140216

**Published:** 2015-10-13

**Authors:** Shin Yup Lee, Jin Eun Choi, Hyo-Sung Jeon, Yi-Young Choi, Won Kee Lee, Eung Bae Lee, Hyun Cheol Lee, Hyo-Gyoung Kang, Seung Soo Yoo, Jaehee Lee, Seung Ick Cha, Chang Ho Kim, Myung Hoon Lee, Young Tae Kim, Sanghoon Jheon, Jae Yong Park

**Affiliations:** 1 Departments of Internal Medicine, School of Medicine, Kyungpook National University, Daegu, Republic of Korea; 2 Departments of Biochemistry and Cell Biology, School of Medicine, Kyungpook National University, Daegu, Republic of Korea; 3 Lung Cancer Center, Kyungpook National University Medical Center, Daegu, Republic of Korea; 4 Biostatistics Center, School of Medicine, Kyungpook National University, Daegu, Republic of Korea; 5 Department of Thoracic Surgery, School of Medicine, Kyungpook National University, Daegu, Republic of Korea; 6 Diagnosis and Prediction Biotechnology, School of Medicine, Kyungpook National University, Daegu, Republic of Korea; 7 Department of Thoracic and Cardiovascular Surgery, Seoul National University School of Medicine, Seoul, Republic of Korea; Istituto dei tumori Fondazione Pascale, ITALY

## Abstract

**Background:**

This study was conducted to investigate whether a panel of eight genetic polymorphisms can predict the prognosis of patients with early stage non-small cell lung cancer (NSCLC) after surgical resection.

**Materials and Methods:**

We selected eight single nucleotide polymorphisms (SNPs) which have been associated with the prognosis of lung cancer patients after surgery in our previous studies. A total of 814 patients with early stage NSCLC who underwent curative surgical resection were enrolled. The association of the eight SNPs with overall survival (OS) and disease-free survival (DFS) was analyzed.

**Results:**

The eight SNPs (*CD3EAP* rs967591, *TNFRSF10B* rs1047266, *AKT1* rs3803300, *C3* rs2287845, *HOMER2* rs1256428, *GNB2L1* rs3756585, *ADAMTSL3* rs11259927, and *CD3D* rs3181259) were significantly associated with OS and/or DFS. Combining those eight SNPs, we designed a prognostic index to predict the prognosis of patients. According to relative risk of death, a score value was assigned to each genotype of the SNPs. A worse prognosis corresponded to a higher score value, and the sum of score values of eight SNPs defined the prognostic index of a patient. When we categorized the patients into two groups based on the prognostic index, high risk group was significantly associated with worse OS and DFS compared to low risk group (aHR for OS = 2.21, 95% CI = 1.69–2.88, *P* = 8.0 x 10^−9^, and aHR for DFS = 1.58, 95% CI = 1.29–1.94, *P* = 1.0 x 10^−5^).

**Conclusions:**

Prognostic index using eight genetic polymorphisms may be useful for the prognostication of patients with surgically resected NSCLC.

## Introduction

Lung cancer, specifically non-small cell lung cancer (NSCLC), is the leading cause of cancer deaths worldwide, with an average 5-year survival rate of 16% [1]. Although surgery is the best treatment modality for a potential cure in the early stages of NSCLC, a large proportion of the patients die from disease recurrence. The 5-year survival rate of patients undergoing curative surgery is 73%, 58%, 46%, and 36% for pathologic stages IA, IB, IIA and IIB, respectively [2]. Pathologic stage is the most important predictor of prognosis after surgical resection of NSCLC. However, patients with the same pathologic stage have a different risk of recurrence and death [2]. Therefore, research focuses on prognostic biomarkers for more precise prognostication of patients after surgery [3]. Given that effective adjuvant chemotherapy is available [4, 5], biomarkers to predict recurrence and prognosis after lung cancer surgery is even more important because they may help select subgroups of patients who will benefit from adjuvant treatment.

Genetic polymorphisms have been investigated for the prognostic/predictive biomarkers to guide therapeutic decisions in cancers, including lung cancer [6–8]. For example, patients with advanced lung cancer may have better outcome by certain chemotherapeutic regimens depending on specific genotypes. Alternatively, patients with certain genotypes may have higher risk of poor prognosis after curative resection, and thereby may benefit from adjuvant chemotherapy. During the past several years, our research has focused on single nucleotide polymorphism (SNP) for prognostic biomarkers in lung cancer patients who underwent curative surgical resection. We have found a number of SNPs in genes potentially involved in the development and progression of cancer to be associated with the prognosis of patients with early stage NSCLC after surgery [9–15].

Carcinogenesis is a multistep process characterized by the accumulation of multiple genetic and epigenetic alterations, which results in alterations in cell physiology that collectively dictate malignant growth: self-sufficiency of growth signals, insensitivity to growth-inhibitory signals, evasion of apoptosis, limitless replicative potential, sustained angiogenesis, and tissue invasion and metastasis [16]. Thus, it is unlikely that any single polymorphism would have a dramatic effect on survival outcomes. The combined analysis of a set of polymorphisms in cancer-related genes may amplify the effects of individual polymorphisms and strengthen their predictive power because they may play important roles at certain stages of carcinogenesis, collectively making up the hallmarks of cancer. In this study, we evaluated the association of a panel of eight genetic polymorphisms from our previous studies that are potentially involved in the development and progression of cancer, with the prognosis of lung cancer patients after surgery by introducing a prognostic index using those eight SNPs in combination.

## Materials and Methods

### Study populations

Written informed consent was obtained from all patients prior to surgery at each of the participating institutions and research protocol was approved by the institutional review boards of Kyungpook National University Hospital (KNUH), Seoul National University Hospital (SNUH), and Seoul National University Bundang Hospital (SNUBH). Eight hundred and fourteen patients with pathologic stages I, II, or IIIA (micro-invasive N2) NSCLC who underwent curative surgical resection were enrolled. Among the 814 patients, 334 cases were obtained from the KNUH, 307 cases from SNUH, and 173 cases from SNUBH. All of the patients included in this study were ethnic Koreans. None of the patients received chemotherapy or radiotherapy prior to surgery. The pathologic staging of the tumors was determined according to the International System for Staging Lung Cancer [2].

### Selection of SNPs and genotyping

We selected eight SNPs from our previous studies found to be associated with the prognosis of patients with early stage NSCLC after surgery. Five SNPs (CD3e molecule, epsilon associated protein [*CD3EAP*] rs967591G>A; tumor necrosis factor receptor superfamily, member 10b [*TNFRSF10B*] rs1047266C>T; and v-akt murine thymoma viral oncogene homolog 1 [*AKT1*] rs3803300A>G; complement component 3 [*C3*] rs2287845T>C; and guanine nucleotide binding protein, beta polypeptide 2-like 1 [*GNB2L1*] rs3756585T>G) were selected from our published papers according to potential function and significance [9–11, 15]. Another three SNPs (homer protein homolog 2 [*HOMER2*] rs1256428A>G; a disintegrin-like and metalloprotease domain with thrombospondin type 1-like 3 [*ADAMTSL3*] rs11259927C>T; and CD3d molecule, delta (CD3-TCR Complex) [*CD3D*] rs3181259T>C) were also included although not yet published. The *HOMER2* rs1256428A>G and *ADAMTSL3* rs11259927C>T were significantly associated with the prognosis of lung cancer after surgery, among (258 potentially functional) SNPs in 15q25 region which has been identified as a lung cancer susceptibility locus in genome wide association studies. *CD3D* rs3181259T>C was identified in our study investigating the SNPs in genes encoding cluster of differentiation 3 (CD3) subunits in the T-cell receptor (TCR) complex, which is vital for T-cell development and plays an important role in the recognition of antigens including tumor antigens. Among four SNPs in *CD3D* and *CD3E* promoters, only *CD3D* rs381259T>C was shown to be predictive of prognosis of lung cancer. In the present study, five SNPs (*CD3EAP* rs967591G>A, *TNFRSF10B* rs1047266C>T, *AKT1* rs3803300A>G, *C3* rs2287845T>C, and *GNB2L1* rs3756585T>G) were tested in an increased number of patients compared with their original studies which included 811, 310, 310, 792 and 792 respectively [9–11, 15]. The other three unpublished SNPs were identified using the same patient population of the present study. Genotyping was performed using SEQUENOM’s MassARRAY^®^ iPLEX assay (SEQUENOM Inc., San Diego, CA) or a restriction fragment length polymorphism assay. The SNP identification numbers, base change, minor allele frequencies and p-value for Hardy-Weinberg equilibrium, and log-rank *P* values for overall survival (OS) and disease free survival (DFS) of the eight SNPs are shown in S1 Table.

### Statistical analysis

Differences in the distribution of genotypes according to the clinicopathologic factors of patients were compared using χ^2^ tests. OS was measured from the day of surgery until the date of death from any cause or to the date of the last follow-up. DFS was calculated from the day of surgery until recurrence or death. The survival estimates were calculated using the Kaplan-Meier method. The differences in OS and DFS across different genotypes were compared using the log-rank test. Hazard ratios (HRs) and 95% confidence intervals (CIs) were estimated using multivariate Cox proportional hazards models, with adjustment for age, gender, smoking status, tumor histology, pathologic stage, and adjuvant chemotherapy. We designed a prognostic index to predict the prognosis of patients using a combination of eight SNPs. According to relative risk of death, a score value was assigned to each genotype of the SNPs. A worse prognosis corresponded to a higher score value: in the additive model, 1 was assigned for low risk, 2 for intermediate risk, and 3 for high risk genotype; in the dominant and recessive models, 1 for low risk, and 3 for high risk genotype. The sum of score values of eight SNPs defined the prognostic index of each patient, which ranged 8–20. Using 15 as the cutoff value, we classified patients with the highest approximate tertile of prognostic index into a high risk group. Then we compared OS and DFS between the high (prognostic index ≥15) and low (prognostic index <15) risk groups. All analyses were performed using Statistical Analysis System for Windows, version 9.2 (SAS Institute, Cary, NC, USA).

## Results

### Patient Characteristics and Clinical Predictors

The clinical and pathologic characteristics of the patients and the association with OS and DFS are shown in Table 1. Upon univariate analysis, age (log-rank *P* [*P*_L-R_] for OS = 3.0 x 10^−5^ and *P*_L-R_ for DFS = 0.01) and pathologic stage (*P*_L-R_ for OS = 7.0 x 10^−7^ and *P*_L-R_ for DFS = 4.0 x 10^−12^) were significantly associated with OS and DFS. Sex (*P*_L-R_ for OS = 0.005) and smoking status (*P*_L-R_ for OS = 0.04) were associated with OS. Tumor histology was associated with DFS (*P*_L-R_ for DFS = 0.04).

**Table 1:** Univariate analysis for overall survival and disease-free survival by clinicopathologic features.

		Overall survival			Disease-free survival		
Variables	No. of patients	No. of deaths(%)*	5Y-OSR (%)^†^	Log-rank *P*	No. of events(%)*	5Y-DFSR (%)^†^	Log-rank *P*
Overall	814	245(30.1)	64		402(49.4)	46	
Age, years							
≤ 64	433	109(25.2)	71	3.0×10^−5^	203(46.9)	50	0.01
> 64	381	136(35.7)	55		199(52.2)	40	
Sex							
Male	598	196(32.8)	61	0.005	299(50.0)	46	0.61
Female	216	49(22.7)	72		103(47.7)	46	
Smoking status							
Never	252	66(26.2)	69	0.04	131(52.0)	42	0.63
Ever	562	179(31.9)	62		271(33.3)	48	
Pack-years^‡^							
〈 40	260	78(30.0)	64	0.17	122(46.9)	48	0.35
≥ 40	302	101(33.4)	60		149(49.3)	48	
Histological types							
SCC	369	109(29.5)	64	0.16	162(43.9)	52	0.04
AC	414	122(29.5)	64		221(53.4)	41	
LCC	31	14(45.2)	54		19(61.3)	44	
Pathologic stage							
I	489	115(23.5)	70	7.0x10^-7^	196(40.1)	53	4.0x10^-12^
II-IIIA	325	130(40.0)	55		206(63.4)	34	

Abbreviations: 5Y-OSR, 5-year overall survival rate; 5Y-DFSR, 5-year disease-free survival rate; SCC, Squamous cell carcinoma; AC, Adenocarcinoma; LCC, Large cell carcinoma.

*Row percentage.

^†^5Y-OSR and 5Y-DFSR, proportion of survival derived from Kaplan-Meier analysis.

^‡^In ever-smokers.

### Associations between SNPs and survival outcomes

The 8 SNPs (*CD3EAP* rs967591, *TNFRSF10B* rs1047266, *AKT1* rs3803300, *C3* rs2287845, *HOMER2* rs1256428, *GNB2L1* rs3756585, *ADAMTSL3* rs11259927, and *CD3D* rs3181259) were found to be significantly associated with OS and/or DFS (Table 2 and S1 Fig) when adjusted for age, gender, smoking status, tumor histology, pathologic stage. Adjusted HRs (aHRs) for OS and DFS were 1.68 and 1.32 (*P* = 1.0 x 10^−4^ and 0.01) for *CD3EAP* rs967591 under recessive model, 1.77 and 1.62 (*P* = 0.006 and 0.004) for *TNFRSF10B* rs1047266 under recessive model, 1.35 and 1.17 (*P* = 0.03 and 0.14) for *AKT1* rs3803300 under dominant model, 1.44 and 1.39 (*P* = 0.004 and 0.006) for *C3* rs2287845 under additive model, 1.25 and 1.11 (*P* = 0.02 and 0.15) for *HOMER2* rs1256428 under additive model, 1.33 and 1.15 (*P* = 0.004 and 0.08) for *GNB2L1* rs3756585 under additive model, 1.52 and 1.35 (*P* = 0.006 and 0.02) for *ADAMTSL3* rs11259927 under recessive model, and 1.45 and 1.24 (*P* = 0.006 and 0.05) for *CD3D* rs3181259 under recessive model, respectively.

**Table 2:** Overall survival and disease-free survival according to each genotype.

		Overall survival				Disease-free survival			
Polymorphism/Genotype	No. of cases(%)*	No. of deaths(%)^†^	5Y-OSR (%)^‡^	HR(95%CI)^¶^	*P* ^¶^	No. of events(%)^†^	5Y-DFSR (%)^‡^	HR(95%CI)^¶^	*P* ^¶^
*CD3EAP* rs967591^#^									
GG	194(24.0)	46(23.7)	72			91(46.9)	46		
GA	410(50.8)	110 (26.8)	69	1.12 (0.79–1.58)	0.52	194(47.3)	50	1.05 (0.82–1.36)	0.67
AA	203(25.2)	87(42.9)	47	1.82 (1.27–2.61)	**0.001**	114(56.2)	37	1.37 (1.03–1.80)	**0.03**
Dominant				1.32. (0.95–1.82)	0.10			1.13 (0.89–1.43)	0.31
Recessive				1.68 (1.29–2.20)	**1.0x10** ^**-4**^			1.32 (1.06–1.64)	**0.01**
Additive				1.37 (1.14–1.65)	**0.001**			1.16 (1.01–1.34)	**0.04**
*TNFRSF10B* rs1047266^#^									
CC	438(54.5)	126(28.8)	65			211(48.2)	47		
CT	301(37.4)	85(28.2)	66	0.95 (0.72–1.26)	0.74	144(47.8)	47	1.02 (0.82–1.26)	0.86
TT	65(8.1)	27(41.5)	54	1.74 (1.15–2.64)	**0.009**	40(61.5)	34	1.64 (1.16–2.30)	**0.005**
Dominant				1.07 (0.83–1.38)	0.62			1.10 (0.90–1.35)	0.33
Recessive				1.77 (1.18–2.65)	**0.006**			1.62 (1.17–2.26)	**0.004**
Additive				1.16 (0.95–1.42)	0.14			1.16 (0.99–1.35)	0.06
*AKT1* rs3803300^#^									
AA	306(37.9)	83(27.1)	67			147(48.0)	47		
AG	395(48.9)	132(33.4)	61	1.42 (1.07–1.87)	**0.01**	200(50.6)	45	1.17 (0.94–1.44)	0.16
GG	106(13.1)	28(26.4)	62	1.10 (0.72–1.70)	0.65	52(49.1)	44	1.18 (0.86–1.62)	0.31
Dominant				1.35 (1.03–1.76)	**0.03**			1.17 (0.95–1.43)	0.14
Recessive				0.91 (0.61–1.35)	0.62			1.08 (0.90–1.44)	0.62
Additive				1.15 (0.96–1.38)	0.14			1.11 (0.96–1.28)	0.16
*C3* rs2287845^#^									
TT	592(73.5)	166(28.0)	66			277(46.8)	48		
TC	199(24.7)	67(33.7)	60	1.32 (0.99–1.75)	0.06	109(54.8)	40	1.33 (1.32–4.24)	**0.01**
CC	14(1.7)	8(57.1)	32	2.95 (1.44–6.05)	**0.003**	12(85.7)	13	2.36 (1.32–4.24)	**0.004**
Dominant				1.39 (1.05–1.82)	**0.02**			1.37 (1.11–1.70)	**0.004**
Recessive				2.66 (1.30–5.43)	**0.007**			2.08 (1.16–3.72)	**0.01**
Additive				1.44 (1.13–1.83)	**0.004**			1.39 (1.15–1.68)	**0.006**
*HOMER2* rs1256428									
AA	241(29.9)	62(25.7)	70			112(46.5)	51		
AG	408(50.6)	126(30.9)	62	1.28 (0.94–1.74)	0.11	205(50.3)	44	1.14 (0.90–1.44)	0.27
GG	157(19.5)	55(35.0)	61	1.55 (1.08–2.23)	**0.02**	81(51.6)	42	1.23 (0.92–1.64)	0.16
Dominant				1.35 (1.01–1.81)	**0.04**			1.16 (0.93–1.45)	0.18
Recessive				1.32 (0.98–1.79)	0.07			1.13 (0.89–1.45)	0.33
Additive				1.25 (1.04–1.49)	**0.02**			1.11 (0.96–1.28)	0.15
*GNB2L1* rs3756585^#^									
TT	382(47.2)	95(24.9)	70			174(45.6)	50		
TG	359(44.4)	120(33.4)	60	1.31 (1.00–1.72)	**0.007**	185(51.5)	43	1.11 (0.90–1.36)	0.34
GG	68(8.4)	27(39.7)	54	1.81 (1.18–2.78)	**0.007**	40(58.8)	38	1.37 (0.97–1.93)	0.08
Dominant				1.48 (1.13–1.93)	**0.004**			1.20 (0.98–1.47)	0.08
Recessive				1.62 (1.08–2.43)	**0.02**			1.34 (0.96–1.86)	0.08
Additive				1.33 (1.10–1.62)	**0.004**			1.15 (0.95–1.34)	0.08
*ADAMTSL3* rs11259927									
CC	319(39.7)	83(26.0)	70			147(46.1)	49		
CT	345(42.9)	98(28.4)	66	1.13 (0.84–1.52)	0.41	169(49.0)	47	1.11 (0.89–1.39)	0.36
TT	140(17.4)	59(42.1)	48	1.62 (1.16–2.27)	**0.005**	80(57.1)	37	1.43 (1.09–1.88)	**0.01**
Dominant				1.28 (0.98–1.68)	0.07			1.19 (0.97–1.47)	0.09
Recessive				1.52 (1.13–2.04)	**0.006**			1.35 (1.06–1.73)	**0.02**
Additive				1.27 (1.07–1.50)	**0.007**			1.18 (1.03–1.36)	**0.01**
*CD3D* rs3181259									
TT	148(18.4)	36(24.3)	70			62(41.9)	54		
CT	409(50.9)	113(27.6)	68	1.09 (0.75–1.59)	0.65	197(48.2)	46	1.16 (0.87–1.54)	0.32
CC	247(30.7)	90(36.4)	55	1.55 (1.05–2.29)	**0.03**	134(54.3)	41	1.38 (1.02–1.87)	**0.04**
Dominant				1.24 (0.87–1.77)	0.24			1.24 (1.00–1.52)	**0.05**
Recessive				1.45 (1.12–1.89)	**0.006**			1.24 (1.00–1.53)	**0.05**
Additive				1.28 (1.06–1.55)	**0.01**			1.17 (1.01–1.36)	**0.03**

Abbreviations: 5Y-OSR, 5-year overall survival rate; 5Y-DFSR, 5-year disease-free survival rate; HR, hazard ratio; CI, confidence interval.

*Column percentage.

^†^Row percentage.

^‡^5Y-OSR and 5Y-DFSR, proportion of survival derived from Kaplan-Meier analysis.

^¶^HRs, 95% CIs and their corresponding *P*-values were calculated using multivariate Cox proportional hazard models, adjusted for age, gender, smoking status, tumor histology, and pathologic stage.

^#^These SNPs were selected from our previous studies: rs967591 [9]; rs1047266 [10]; and rs3803300 [11]; rs2287845 and rs3756585 [15]

### Combined analysis of multiple SNPs

**Fig 1:**
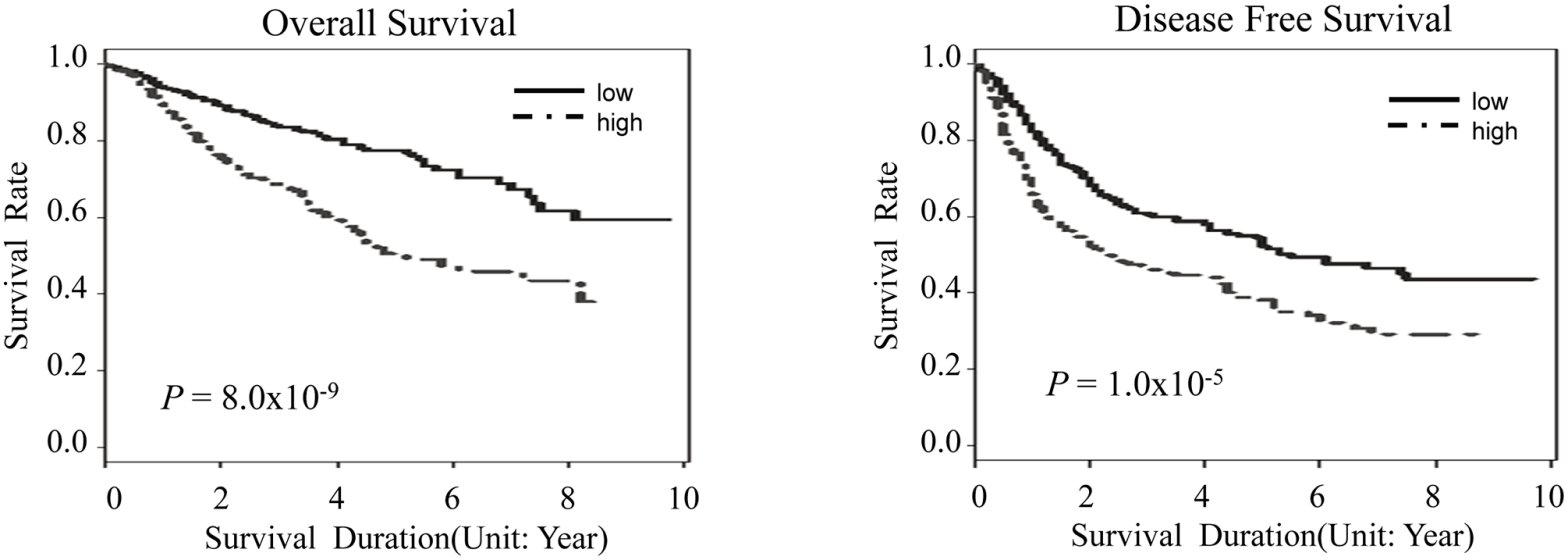
Kaplan-Meier plots of overall survival and disease-free survival according to risk groups. *P* values in the multivariate Cox proportional hazard model.

We performed an exploratory analysis investigating the combined effects of eight SNPs on OS and DFS. We designed a prognostic index as described in the Statistical analysis section. When we categorized the patients into high (prognostic index ≥ 15) and low (prognostic index <15) risk group, high risk patients were significantly associated with worse OS and DFS compared to low risk group at a much higher statistical significance compared with individual SNP analysis (aHR for OS = 2.21, 95% CI = 1.69–2.88, *P* = 8.0 x 10^−9^, and aHR for DFS = 1.58, 95% CI = 1.29–1.94, *P* = 1.0 x 10^−5^, Table 3 and Fig 1).

**Table 3:** Combined effect of 8 SNPs on overall survival and disease-free survival.

		Overall survival					Disease-free survival				
Risk group (Prognostic index^#^)	No. of cases(%)*	No. of deaths(%)^†^	5Y-OSR (%)^‡^	*P*_L-R_	HR(95%CI)^¶^	P^¶^	No. of events(%)^†^	5Y-DFSR (%)^‡^	*P*_L-R_	HR(95%CI)^¶^	*P* ^¶^
All patients											
Low risk (<15)	468(60.6)	98(20.9)	76	1.0x10^-9^	1.00		200 (42.7)	52	4.0x10^-6^	1.00	
High risk (≥15)	304(39.4)	127(41.8)	50		2.21 (1.69–2.88)	8.0x10^-9^	178 (58.6)	37		1.58 (1.29–1.94)	1.0x10^-5^

Abbreviations: 5Y-OSR, 5-year overall survival rate; 5Y-DFSR, 5-year disease-free survival rate; *P*_L-R_, log-rank *P*; HR, hazard ratio; CI, confidence interval.

*Column percentage.

^†^Row percentage.

^‡^5Y-OSR and 5Y-DFSR, proportion of survival derived from Kaplan-Meier analysis.

^¶^HRs, 95%CIs and their corresponding *P*-values were calculated using multivariate Cox proportional hazard models, adjusted for age, gender, smoking status, tumor histology, and pathologic stage.

^#^For additive genetic model, score value 1 assigned for low risk, 2 for intermediate risk, 3 for high risk genotype among WW, WV and VV genotypes; For dominant genetic model, 1 for low risk, 3 for high risk genotype among WW and WV+VV genotypes; For recessive genetic model, 1 for low risk, 3 for high risk genotype among WW+WV and VV genotypes (W, wild allele; V, variant allele)

## Discussion

This study was conducted to investigate whether a panel of eight genetic polymorphisms could predict the prognosis of patients with early stage NSCLC after surgical resection. We selected eight SNPs which were associated with the prognosis of lung cancer patients after surgery from our previous studies, and evaluated those SNPs in 814 patients. The eight SNPs (*CD3EAP* rs967591, *TNFRSF10B* rs1047266, *AKT1* rs3803300, *C3* rs2287845, *HOMER2* rs1256428, *GNB2L1* rs3756585, *ADAMTSL3* rs11259927, and *CD3D* rs3181259) were significantly associated with OS and/or DFS. Combining those eight SNPs, we designed a prognostic index to predict the prognosis of patients. After categorizing patients into high and low risk groups by the prognostic index, we found the high risk group was significantly associated with worse OS and DFS than the low risk group. Combined analysis using the prognostic index had a much better resolution in predicting the prognosis of patients compared to individual SNP analysis. The prognostic index using those eight genetic polymorphisms may be useful for identifying patients at a higher risk of disease recurrence and death after surgical resection of NSCLC, and thereby help to select patients for adjuvant chemotherapy.

The most important finding of the present study is that the prognostic index based on the eight genetic polymorphisms may lead to a significantly better prediction of survival compared to individual SNP analysis in early-stage NSCLC after surgery. Given that carcinogenesis is a multi-step process characterized by the accumulation of multiple genetic and epigenetic alterations which collectively determine malignant phenotype, it is unlikely that any single polymorphism could be a powerful predictor of survival outcome. In addition, using a relatively large number of patients, we partly replicated our previous studies of the individual SNPs and patient survival association. Functional consequences of the SNPs from our previous studies further supports the plausibility of the current results.

The *CD3EAP* encodes a nucleoprotein, and is positioned in an anti-sense orientation to, and overlaps with, *ERCC1*. Although the biological function of the CD3EAP is unclear, the protein may be a member of the RNA polymerase I transcription complex that synthesize ribosomal RNA precursors, thus implicating CD3EAP in cell proliferation [17]. In addition, CD3EAP isoform 2 interacts with the CD3 epsilon subunit molecule of TCR-CD3 complex. In our previous study, functional analysis suggested that the inherited rs967591G>A affects *CD3EAP* expression [9]. TNFRSF10B (DR5) is one of the TNFSF10 (TRAIL) receptors and initiates TRAIL-mediated apoptosis. DR5 is expressed in a variety of cancers, including NSCLC, and its expression has been linked to survival outcomes in many types of cancer [18–20]. AKT plays a pivotal role in the phosphatidylinositol 3-kinase (PI3K)-related signaling pathway, regulating cell survival, proliferation, and anti-apoptosis [21, 22]. Furthermore, AKT is implicated in the regulation of angiogenesis and metastasis, two important processes in cancer development and progression [23, 24]. Aberrantly activated AKT expression has been reported and linked to the prognosis of patients with lung cancer [22, 25, 26].

The complement system has a major role in innate and adaptive immunity. The *C3* protein is central to the activation of all the three complement pathways, the classical, alternative, and mannose-binding lectin pathways [27, 28]. It has been reported that the complement system is activated in various types of cancer, including lung cancer [28–30]. Although complements have been thought to participate in immunosurveillance against tumors [28], there is growing evidence that complements play oncogenic roles in tumorigenesis [31, 32]. Homer family proteins are known as post-synaptic adaptor proteins that interact with several proteins, such as metabotropic glutamate receptors, inositol 1,4,5-triphosphate receptors, and modulate the Ca2+ signaling pathway in neurons [33]. HOMER2 interacts with the C-terminal region of MYO18B, a candidate tumor suppressor gene involved in the pathogenesis of human cancers including lung cancer [33]. It was reported that the MYO18B gene is hemizygously deleted in 60% and mutated in 15% of lung cancers, and that reduced expression of MYO18B, often accompanied by promoter DNA methylation and histone deacetylation, was observed in 70% of lung cancers [34, 35]. It has been reported that coexpression of HOMER2 with MYO18B enhanced the ability of MYO18B to suppress the anchorage-independent growth of a human lung cancer cell line, suggesting that HOMER2 and MYO18B cooperate in tumor suppression [33].

*GNB2L1*, alias *RACK1*, belongs to a WD40 protein family that includes the β subunit of G-proteins. As a scaffold protein, GNB2L1 interacts with signaling molecules such as cyclic AMP-specific phosphodiesterase 4D isoform 5 (PDE4D5), the SRC family of tyrosine kinases, and β integrins, as well as PKC, and thus plays a pivotal role in a wide range of biologic responses, including cell growth, adhesion, and migration [36–38]. Studies have indicated that GNB2L1 plays an important role in cancer progression and that its expression is up-regulated during angiogenesis in some types of cancers, including lung cancer [39–41]. In addition, *GNB2L1* over-expression has been strongly associated with poor clinical outcomes of cancer patients [41, 42]. According to our unpublished data, *in vitro* promoter assay and electrophoretic mobility shift assay (EMSA) revealed that the rs3756585 T-to-G change increased transcription factor binding and promoter activity of *GNB2L1*.

*ADAMTSL3* encodes a secreted glycoprotein with strong similarity to members of the ADAMTS (a disintegrin and metalloproteinase with thrombospondin motif) family. The ADAMTS family has been involved in various human biological processes (normal or pathological), including connective tissue structure, cancer, coagulation, arthritis, angiogenesis and cell migration [43]. They are involved in cancer-related processes such as proliferation (e.g. the cleavage of epithelial growth factor (EGF) family signal protein precursors, including EGF and tumor growth factor-α), apoptosis, angiogenesis, and in the destruction of components of the extracellular matrix, which facilitate invasion and metastasis [44]. ADAMTS-like proteins, including ADAMTSL3, lack proteolytic activity typical for ADAMTS family proteins, but appear to have important regulatory roles in the extracellular matrix [43]. Recently, frequent mutations in *ADAMTSL3* have been identified recently in colorectal cancer [45]. T-cells play an important role in the immune response. The TCR is responsible for the recognition of antigens including tumor antigens bound to major histocompatibility complex (MHC) molecules. TCR complexes contain a TCR heterodimer and four CD3 subunits: CD3-gamma, -delta, -epsilon and -zeta. The CD3 complex is vital for T cell development as well as T cell function [46]. Accumulating evidences indicate that immune cells play important roles in development and progression of cancer depending on their mode of differentiation and cytokine signaling in the tumor microenvironment. CD8+ cytotoxic T lymphocytes, CD4+ T helper (TH) 1 cells and natural killer (NK) cells function as major antitumor effector cells, whereas CD4+ TH2 cells, myeloid-derived suppressor cells (MDSCs) seem to promote tumor progression [47].

In this study, we showed that prognostic index based on eight genetic polymorphisms was an independent predictive factor for the survival of patients with NSCLC after surgery. The prognostic index may help to predict patients’ prognoses more precisely when used in addition to pathologic stage, the single most powerful predictor of prognosis after surgical resection of NSCLC, therefore being particularly useful in selecting patients who may benefit from adjuvant chemotherapy. Because the current study partly replicated our previous studies on the association between the eight SNPs and patient survival using a relatively large number of patients, reduced chance of false positive associations is expected. However, the prognostic index needs to be further tested in future prospective studies including clinical trials. In addition, further studies are needed to understand the roles of those genes in lung cancer and to clarify the association between the SNPs and prognosis.

In conclusion, this study shows that a panel of eight SNPs could be useful to identify patients with a higher risk of disease recurrence and death after surgical resection of NSCLC, and thereby help to select patients for adjuvant chemotherapy. Further studies are required to confirm the validity of these SNPs in other ethnic populations.

## Supporting Information

S1 Table Summary of the selected and genotyped SNPs and the survival outcomes. (DOCX)

S1 Fig Kaplan-Meier plots of overall survival and disease-free survival according to genotypes. *CD3EAP* rs967591G>A, A); *TNFRSF10B* rs1047266 C>T, B); *AKT1* rs3803300 A>G, C); *C3* rs2287845T>C, D); *HOMER2* rs1256428 G>A, E); *GNB2L1* rs3756585 T>G, F); *ADAMTSL3* rs11259927 C>T, G); and *CD3D* rs3181259 C>T, H). *P* values by Log-rank test.
(PPTX)
