# Supplementary material for: A Panel of Genetic Polymorphism for the Prediction of Prognosis in Patients with Early Stage Non-Small Cell Lung Cancer after Surgical Resection
Source: PLoS One. 2015 Oct 13;10(10):e0140216. doi: 10.1371/journal.pone.0140216 (PMC4603900; doi:10.1371/journal.pone.0140216)
Supplement: S1 Table — (DOCX) [file pone.0140216.s001.docx]

S1 Table. Summary of the selected and genotyped SNPs and the survival outcomes.

| Polymorphism | |  | Genotype | |  | Log-Rank *P* for Overall survival | | |  | Log-Rank *P* for Disease-free survival | | |
| --- | --- | --- | --- | --- | --- | --- | --- | --- | --- | --- | --- | --- |
| ID No. | Base change |  | MAF | HWE *P* |  | Referent | Dominant | Recessive |  | Referent | Dominant | Recessive |
| *CD3EAP* rs967591 | G/A |  | 0.49 | 0.67 |  | 9.0x10^-6^ | 0.04 | 1.0x10^-5^ |  | 0.05 | 0.26 | 0.02 |
| *TNFRSF10B* rs1047266 | C/T |  | 0.27 | 0.19 |  | 0.02 | 0.51 | 0.004 |  | 0.01 | 0.36 | 0.003 |
| *AKT1* rs3803300 | A/G |  | 0.38 | 0.22 |  | 0.09 | 0.06 | 0.58 |  | 0.57 | 0.29 | 0.85 |
| *C3* rs2287845 | T/C |  | 0.14 | 0.56 |  | 0.001 | 0.03 | 0.001 |  | 5.0x10^-5^ | 0.004 | 0.001 |
| *HOMER2* rs1256428 | A/G |  | 0.45 | 0.50 |  | 0.07 | 0.07 | 0.05 |  | 0.41 | 0.28 | 0.27 |
| *GNB2L1* rs3756585 | T/G |  | 0.31 | 0.20 |  | 0.02 | 0.01 | 0.08 |  | 0.14 | 0.09 | 0.14 |
| *ADAMTSL3* rs11259927 | C/T |  | 0.39 | 0.01 |  | 0.01 | 0.11 | 0.003 |  | 0.04 | 0.16 | 0.01 |
| *CD3D* rs3181259 | T/C |  | 0.44 | 0.35 |  | 0.03 | 0.01 | 0.13 |  | 0.05 | 0.04 | 0.05 |

Abbreviation: MAF, minor allele frequency; HWE *P*, *P* value for Hardy-Weinberg equilibrium.
