# Supplementary material for: A Panel of Genetic Polymorphism for the Prediction of Prognosis in Patients with Early Stage Non-Small Cell Lung Cancer after Surgical Resection
Source: PLoS One. 2015 Oct 13;10(10):e0140216. doi: 10.1371/journal.pone.0140216 (PMC4603900; doi:10.1371/journal.pone.0140216)
Supplement: S1 Fig — CD3EAP rs967591G>A, A); TNFRSF10B rs1047266 C>T, B); AKT1 rs3803300 A>G, C); C3 rs2287845T>C, D); HOMER2 rs1256428 G>A, E); GNB2L1 rs3756585 T>G, F); ADAMTSL3 rs11259927 C>T, G); and CD3D rs3181259 C>T, H). P values by Log-rank test. (PPTX) [file pone.0140216.s002.pptx]

## Slide 1
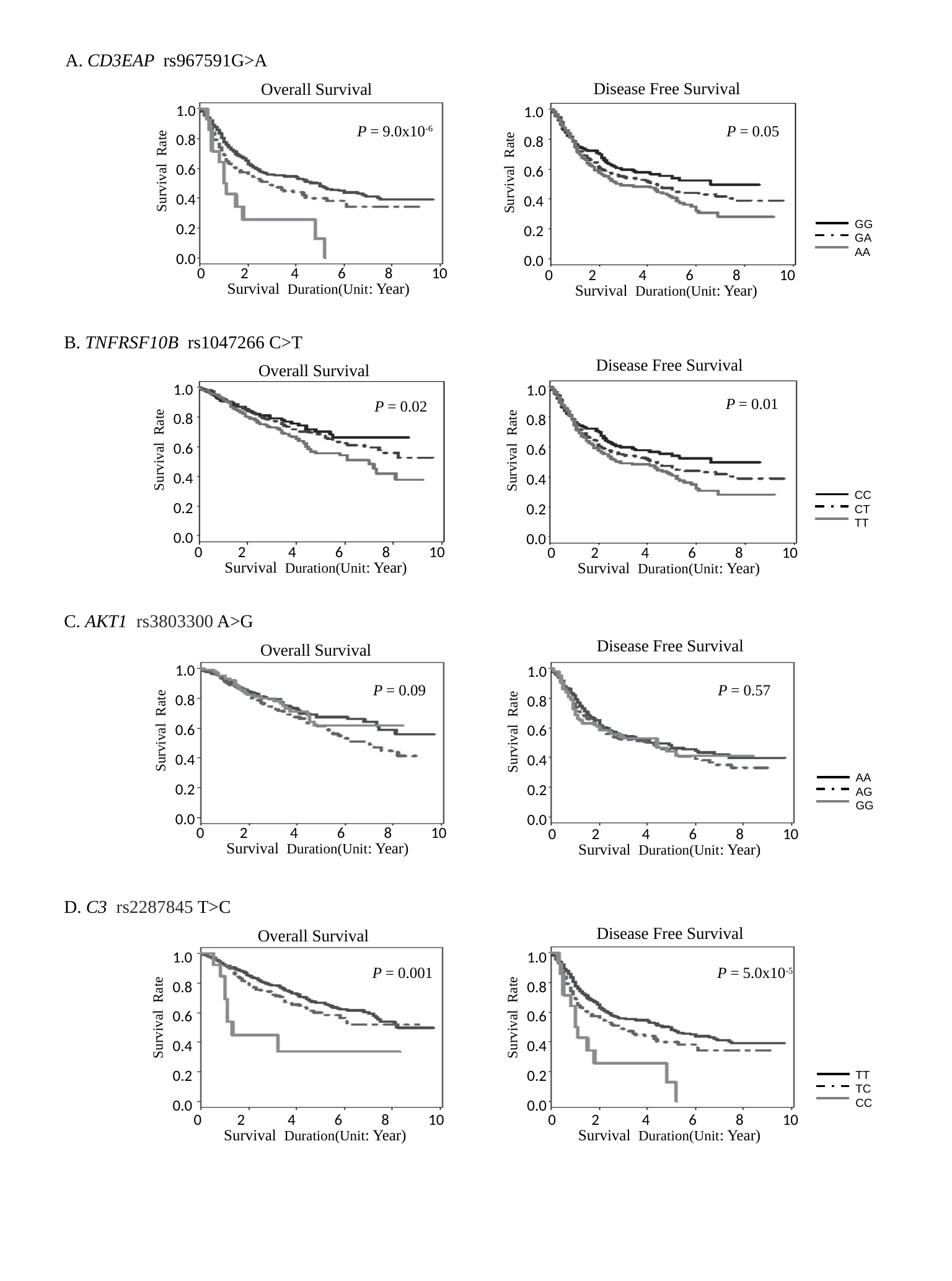

A. CD3EAP rs967591G>A
Overall Survival
Disease Free Survival
1.0
0.8
0.6
0.4
0.2
0.0
1.0
0.8
0.6
0.4
0.2
0.0
Survival Rate
Survival Rate
P = 9.0x10^-6^
P = 0.05
GG
GA
AA
0 2 4 6 8 10
0 2 4 6 8 10
Survival Duration(Unit: Year)
Survival Duration(Unit: Year)
B. TNFRSF10B rs1047266 C>T
Overall Survival
1.0
0.8
0.6
0.4
0.2
0.0
Survival Rate
0 2 4 6 8 10
Survival Duration(Unit: Year)
Disease Free Survival
1.0
0.8
0.6
0.4
0.2
0.0
Survival Rate
0 2 4 6 8 10
Survival Duration(Unit: Year)
P = 0.01
P = 0.02
CC
CT
TT
C. AKT1 rs3803300 A>G
Overall Survival
1.0
0.8
0.6
0.4
0.2
0.0
Survival Rate
0 2 4 6 8 10
Survival Duration(Unit: Year)
Disease Free Survival
1.0
0.8
0.6
0.4
0.2
0.0
Survival Rate
0 2 4 6 8 10
Survival Duration(Unit: Year)
P = 0.09
P = 0.57
AA
AG
GG
D. C3 rs2287845 T>C
Overall Survival
1.0
0.8
0.6
0.4
0.2
0.0
Survival Rate
0 2 4 6 8 10
Survival Duration(Unit: Year)
Disease Free Survival
1.0
0.8
0.6
0.4
0.2
0.0
Survival Rate
0 2 4 6 8 10
Survival Duration(Unit: Year)
P = 0.001
P = 5.0x10^-5^
TT
TC
CC

## Slide 2
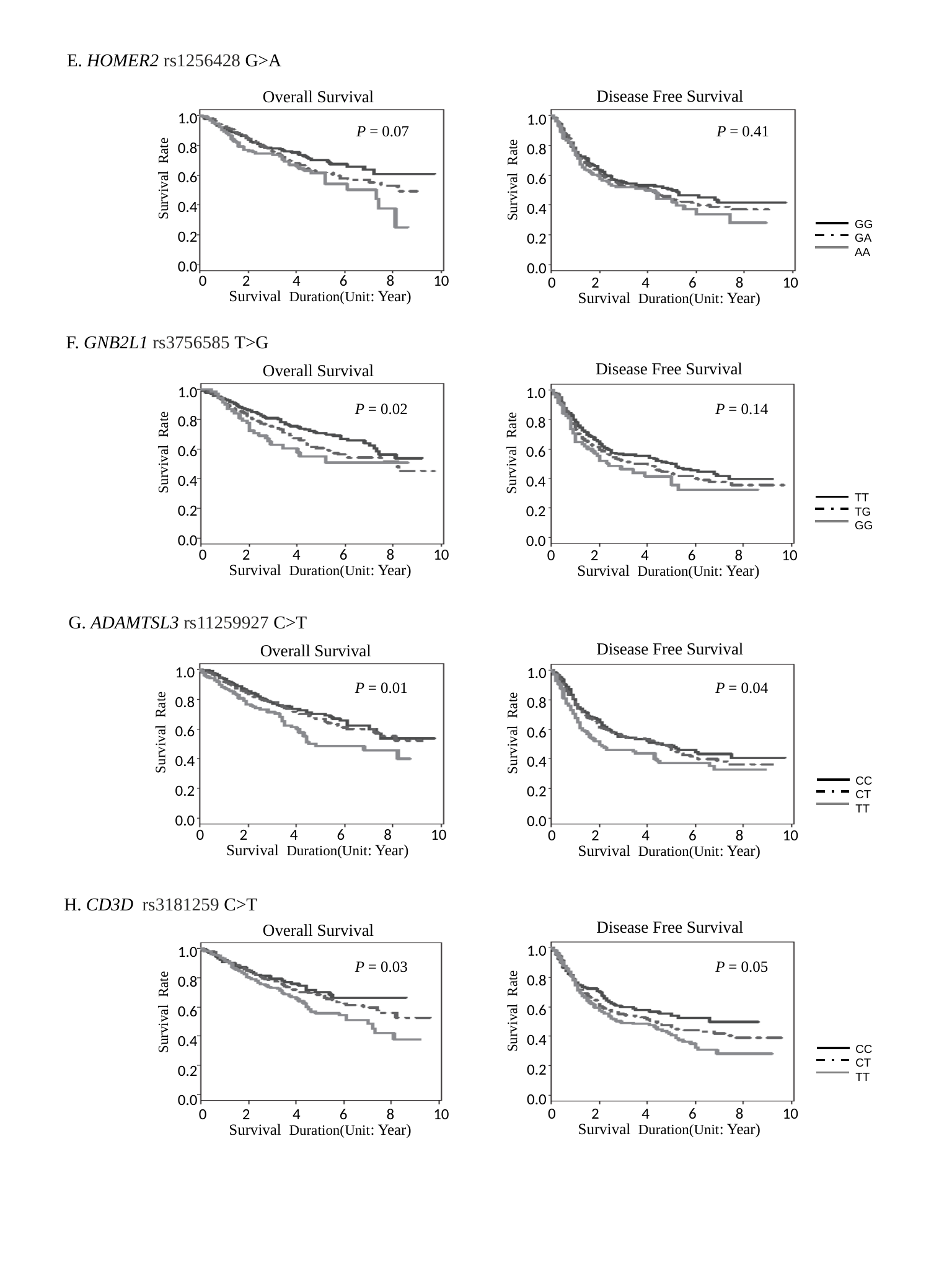

E. HOMER2 rs1256428 G>A
Overall Survival
1.0
0.8
0.6
0.4
0.2
0.0
Survival Rate
0 2 4 6 8 10
Survival Duration(Unit: Year)
Disease Free Survival
1.0
0.8
0.6
0.4
0.2
0.0
Survival Rate
0 2 4 6 8 10
Survival Duration(Unit: Year)
P = 0.07
P = 0.41
GG
GA
AA
F. GNB2L1 rs3756585 T>G
Overall Survival
1.0
0.8
0.6
0.4
0.2
0.0
Survival Rate
0 2 4 6 8 10
Survival Duration(Unit: Year)
Disease Free Survival
1.0
0.8
0.6
0.4
0.2
0.0
Survival Rate
0 2 4 6 8 10
Survival Duration(Unit: Year)
P = 0.02
P = 0.14
TT
TG
GG
G. ADAMTSL3 rs11259927 C>T
Overall Survival
1.0
0.8
0.6
0.4
0.2
0.0
Survival Rate
0 2 4 6 8 10
Survival Duration(Unit: Year)
Disease Free Survival
1.0
0.8
0.6
0.4
0.2
0.0
Survival Rate
0 2 4 6 8 10
Survival Duration(Unit: Year)
P = 0.01
P = 0.04
CC
CT
TT
H. CD3D rs3181259 C>T
Overall Survival
1.0
0.8
0.6
0.4
0.2
0.0
Survival Rate
0 2 4 6 8 10
Survival Duration(Unit: Year)
Disease Free Survival
1.0
0.8
0.6
0.4
0.2
0.0
Survival Rate
0 2 4 6 8 10
Survival Duration(Unit: Year)
P = 0.03
P = 0.05
CC
CT
TT

## Slide 3
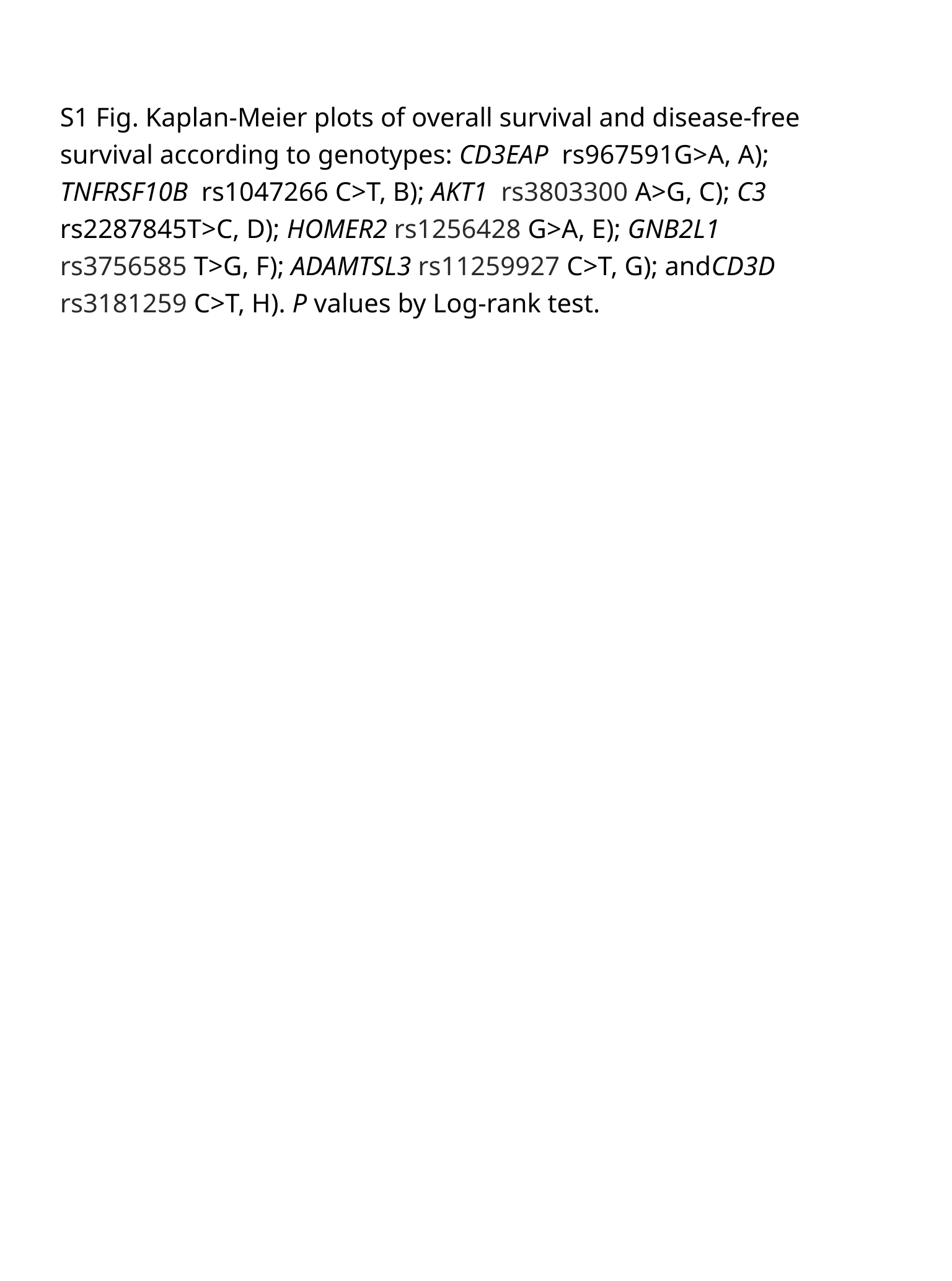

S1 Fig. Kaplan-Meier plots of overall survival and disease-free survival according to genotypes: CD3EAP rs967591G>A, A); TNFRSF10B rs1047266 C>T, B); AKT1 rs3803300 A>G, C); C3 rs2287845T>C, D); HOMER2 rs1256428 G>A, E); GNB2L1 rs3756585 T>G, F); ADAMTSL3 rs11259927 C>T, G); andCD3D rs3181259 C>T, H). P values by Log-rank test.
